# Understanding Plant Parasitism: *Hydnora Africana* and European Scientific Practice in the Early Nineteenth Century**

**DOI:** 10.1002/bewi.70032

**Published:** 2026-08-23

**Authors:** Katherine Arnold

**Affiliations:** ^1^ School of Histories, Languages and Cultures University of Liverpool 8‐12 Abercromby Square Liverpool L69 7WZ UK

**Keywords:** boundary object, nineteenth century, parasitism, plants, professionalization, scientific practice, southern Africa, taxonomy

## Abstract

This article will analyze the uncertainties around collecting and classifying *Hydnora africana* in the first half of the nineteenth century. Emerging from the distinctive dryland ecologies of South Africa, *Hydnora* was interpreted as strange and cryptic by Europeans primarily because it is a root parasite—a complex floral trait that was little understood. Its unique timescale, dramatic structural change in drying, and resistance to travel made collection extremely difficult,and an inability to collect such plants informed debates on how naturalists could make classifications without seeing them *in situ* and what characteristics should be prioritized when ordering flowering plants. Attempts at understanding parasitism, therefore, became possible only through global comparisons to other parasitic plants in places like Sumatra, Spain, and the Mediterranean. This created anxieties around authority between the lab and the field, and amateur and professional, testing European scientific methods at the crucial moment in which natural history was fragmenting into disciplines and Linnaean taxonomy became increasingly ineffective. This article thus uses *Hydnora africana* as a boundary object between shifting geographies, institutions, and epistemologies, demonstrating how the parasitism debate embodied all sorts of questions related to control—over people, places, the natural world—but especially the limits of European knowledge systems.

1

At the 1836 meeting of the *Gesellschaft Deutscher Naturforscher und Ärzte* in Jena, Gustav Kunze of Leipzig University presented a dried specimen of *Hydnora africana*, “a remarkable Asarine which grows parasitic on the large Euphorbias in the Carro [*sic*] near Worcester” in the Western Cape region of South Africa.^1^ Kunze's identification of *Hydnora* as part of the *Asarina* genus would, however, be short‐lived, rooting us in a particular moment of European knowledge production in the first half of the nineteenth century. Though it was common for naturalists and collectors to move plants around ever‐shifting taxonomies, particularly unknown plants from unfamiliar colonial environments, *Hydnora* posed a singular challenge to developing botanical taxonomies because of its most characteristic attribute: parasitism. This complex floral trait was little understood, testing the accepted methods of European science at a crucial moment in which natural history was beginning to fragment into distinct disciplines, the Linnaean system became increasingly ineffective at categorizing flowering plants, and questions arose about who held authority in science and where it should be practiced.^2^ This paper thus intends to use *Hydnora africana* as a “boundary object” to analyze the significance of (and uncertainties around) taxonomic debates, and how difficult traits such as parasitism helped to challenge established epistemic orders. Drawing on a range of methodologies, it aims to understand the intersecting layers that helped to construct meaning around the plant and its characteristic parasitism. Not meant to be a comprehensive object biography of *Hydnora*, it instead puts material at the center of complex imperial, professional, and cultural networks to demonstrate the sweeping changes taking place in the European scientific world. Fundamentally, it is a story about power—over people, places, knowledges, and the natural world—in a crucial period of imperial expansion.

Scholars across disciplines have found it productive to work with boundary objects as a “means of translation” between different social worlds, scopes, and scales.^3^ Susan Leigh Star and James Griesemer first introduced the concept within the framework of a zoology museum, where a wide variety of human and non‐human actors converged and were forced to organize around a shared objective.^4^ In their analysis they demonstrated how particular objects or things—whether they be specimens, directories, classifications, maps, or methods—shaped the way knowledge was collected, managed, and coordinated.^5^ Importantly, they take an “ecological analysis” which “does not presuppose an epistemological primacy for any one viewpoint.”^6^ We see Bruno Latour at work here in their concern with the flow of objects and concepts through the network of participating actors and social worlds.^7^ As these ideas have developed, scholars have traced not just physical movements of objects, but also their contested meanings and interpretations, how they altered classificatory schema or theoretical frameworks, and how they were debated in public or private settings.^8^ With this in mind, this paper intends to follow *Hydnora africana* through different imperial and national units, through the perspectives of varied amateurs and professionals, and in a range of field and institutional contexts, all of which permanently and often violently divorced *Hydnora* from its original ecological and cultural landscape.^9^

In the environmental humanities and STS, scholars have most often turned to Latour's actor‐network‐theory to show not just how objects had “lives” or acted as “boundary objects,” but rather were active agents in the making of history.^10^ Though there are still some unresolved questions as to whether we can truly consider living beings or material phenomena as having agency, Jane Bennett has argued that we should instead think of it as a form of “creativity.”^11^ In her view, things “can create order or disorder with or without intentionality” and that willpower (central to human agency) can be understood as a response to linguistic, cultural, biological, or ecological factors.^12^ This has been mostly applied to vertebrates with a relative neglect of plants.^13^ There is an ostensible plant blindness not just in scholarly work but in our everyday lives; however, recently there has been an emergence of a small, vibrant interdisciplinary outlet for research into the significance of plants.^14^ Some have argued that “vegetal time” is key to the idea of plant agency. It allows us to explore the “peculiar and multiple temporalities of plants” which may well act as an ecological “locus of resistance.”^15^ We can use these ideas to think through *Hydnora*. Because of its fleshy body and infrequent flowering time, it tended to evade the usual processes of locating, extracting, drying, shipping, classifying, and displaying plants and plant specimens that had been standardized in Europe.^16^ Thus, it was essentially protected by the limits of the environment in which it lived and by the very nature of its parasitism. While some might argue that this is simply a case of certain species or traits not fitting into the very restricted, human‐made systems of classification, this paper hints at *Hydnora* having agency: how some aspects of its materiality unintentionally led to confusion amongst European collectors and botanists in their attempts to make sense of an unfamiliar plant.

This paper touches on some well‐trodden ground in its consideration of the relationship between the field and the lab, the professionalization of the sciences, and the coloniality of botanical practice.^17^ It contributes to the politics of authority at a time of fraught tension between the long‐standing use of Linnaean taxonomy versus the growing acceptance of the natural system. Part of that challenge had to do with whether naturalists could understand difficult plant characteristics only through dried specimens or illustrations, particularly when access to live plants was hard to organize, demonstrating the contested nature of what counted as reliable evidence in processes of naming plants. European botanists were unable to cope with the sheer variety of plant forms arriving from across the world, and thus it became necessary to make locally collected botanical specimens meaningful by placing them into a broader, global context. Plants were not neutral objects, but rather essential components of the ideology, language, and practice of empire.^18^ Parasitism, too, is a loaded concept, part of a complex system of racialization that was justified through science.^19^ Working in this difficult period of unprecedented epistemological change, using the “boundary object” concept allows us to cut through some of the difficulties this history and historiography present. By following *Hydnora* and the parasitism debate through this moment, it reveals things we might not normally see in a paper on this topic: the importance of commercial collecting, the centrality of naturalists in the German states, and a slew of plants that became important to the consolidation of taxonomy in nineteenth‐century science.

Finally, this paper places the under‐appreciated flora of southern Africa into the center of a story about the development of the global sciences. A long‐standing interest in, and circulation of, Cape flora since the seventeenth century meant that by the first decades of the nineteenth century, attention to Cape plants had declined. As Robert E. Kohler maintains, museums, nurseries, and gardens would decide they had enough material from one region and shift their attention to other parts of the world, even if those regions had not yet been exhaustively explored.^20^ Instead, focus shifted to things like orchids (resulting in *orchidelirium*), ferns (*pteridomania*), the giant *Victoria amazonica* water lily from Brazil, and the *Rafflesia arnoldii* from the mountainous jungles of Sumatra.^21^ The growing significance of biogeography also played a role in determining which landscapes were privileged in botanical collecting and research.^22^ Thus, naturalists rarely dwelled on the landscapes of southern Africa, paying little attention to the *Proteas*, *Ericas*, and *Pelargoniums* that had once been popular in the gardens of Europe, let alone to the seemingly unexciting shrubs, aloes, and succulents that make up the distinct *fynbos* vegetation of the region. Indeed, historiography has also tended to favor tropical and mountainous ecosystems and the strange plants that emerged from them, forgetting that the Cape Floristic Region—the smallest of the six recognized floral kingdoms—has played a hugely significant role in debates on the development of biodiversity as a concept.^23^ This paper attempts to write one aspect of southern Africa's vegetal landscape back into histories of science, using non‐human sources to tell stories that are not well represented in the archive or in our current histories.

## 2 Collection in the Field

Due to its unique evolutionary development, the Cape Floristic Region is remarkable for its diversity and density of endemic species and range of unusual plant adaptations, complemented by an equally distinct set of insects, fauna, marine life, and topography essential to the ecological harmony of the biome.^24^ Within South Africa alone there are ten families of plants that include parasites, ranging from the more unassuming *Loranthaceae* and *Viscaceae* (mistletoes) to grotesque extremes like *Hydnoraceae* and *Balanophorceae*.^25^ Called *kannip* in Khoekhoegowab and found in the semi‐arid regions of the northwestern Cape and southern Namibia, *Hydnora* is a root holoparasite.^26^ Growing completely subterranean, it feeds directly onto species of succulent *Euphorbia* by invading the host's roots through parasitic structures called haustoria.^27^ It is incapable of photosynthesis and thus contains no chlorophyll.^28^ After leaching enough energy from its spurge host, a fleshy orange‐pink flower emerges, releasing a fetid odor to attract its carrion and dung beetle pollinators.^29^ The Boer farmers who lived in the area called it “Jackhalls Kost” (*jakkalskos*), or jackal food, and the fruit is thought to have a sweet and starchy taste.^30^ In 1856, Cape colonist William Guybon Atherstone wrote that the “fruit is very good and is keenly hunted for by Bushmen and wild pigs, jackals and many other animals. I have frequently eaten it in Namaqualand where it attains the size of a small orange.”^31^ The rhizophores of *Hydnora*, called *uMavumbuka* or “the one that pops up” in isiZulu, are used as traditional medicine in South Africa to treat diarrhea, piles, acne, menstrual problems, stomach cramps, inflammation of the throat, and to stop bleeding, perhaps a result of the plant's high tannin content.^32^ It seems *Hydnora* regularly featured in the diets and medicinal practices of the Khoekhoe, San, and other African groups who had long co‐existed with it.

Yet, the Europeans who first encountered *Hydnora* believed it to have a cryptic nature and uncommon appearance, likely due to its infrequent flowering and particular materiality. It was first collected by Swedish naturalist Carl Peter Thunberg from the Bokkeveld Mountains in the Hantam district in 1774 when the Cape Colony was still under Dutch control.^33^ Since its founding by the Dutch East India Company in 1652, the Cape Colony was a significant maritime and scientific hub for Europeans traveling to and from the East Indies because of its mild climate and well‐known botanic garden. On *Hydnora*, Thunberg remarked, “so strange is its composition that many would certainly doubt the existence of such a plant on the face of the earth.”^34^ At this point in the development of the Linnaean taxonomic system, there was no obvious place for *Hydnora*. Instead, Thunberg placed it into a broader category based on its morphology, assigning it as a fungus related to the genus *Hydnum* both in his published travels and in an initial description written for the Swedish Royal Academy of Sciences.^35^ The same classification was made by the last of the Linnaean disciplines, Erik Acharius, who published a dissertation entitled *Planta Aphyteja* based on the specimen Thunberg sent to Uppsala.^36^ He, too, used the tools available to him in the Linnaean system and came to the same conclusion. The assumption that *Hydnora* was a fungus took some time to disprove, as it was only distinguishable from fungi when the flower was open. This was a common problem when collecting plants out of season or which had peculiar temporalities; the Linnaean classification system was fundamentally based on the flower, making identification tricky with species that had irregular flowering times.

Though the Cape was a popular destination for naturalists to collect between the European voyages of discovery and the end of the Napoleonic Wars, we do not really see *Hydnora* appear much in European scientific literature until the 1820s.^37^ By then, the British held political control and a new generation of Cape collectors embarked on collecting expeditions within and beyond the colony, collecting rare *Hydnora* specimens to sell commercially in Europe. These collectors—Christian Ecklon, Karl Zeyher, and Johann Franz Drège—all came from artisanal backgrounds in the German states and financed their own expeditions, supporting themselves through the formation of natural history businesses predicated on the sale of specimens.^38^ Ecklon had trained as an apothecary, working for five years at the Pallas and Polemann pharmacy in Cape Town with Drège's brother, Carl Friedrich.^39^ Zeyher and Drège, on the other hand, were trained horticulturalists. While Zeyher apprenticed with his uncle in the ducal gardens of Schwetzingen in the state of Baden, Drège had previously held positions in the gardens of Munich, Berlin, St. Petersburg, and Riga. Their backgrounds taught them not only how to interpret the natural world, but also to display and sell it. They were sharply attuned to the commercial possibilities the natural world offered, understanding the market for exotic specimens as (allegedly) exceptionally lucrative.^40^ Both groups created their own (competing) natural history businesses predicated on the necessity of thinking of their materials, specimens, and African labor in terms of economic value. These so‐called entrepreneurial collectors were not necessarily interested in making imperial nature governable, they merely wanted to make it marketable. Their focus would variously shift between the desires of the market and the demands of science, making classification all the more important as a wider network of practitioners became entangled in the processes of naming plants. They therefore occupied an “ambiguous and sometimes problematic position” within the cultural economy of natural history networks.^41^ From the 1820s to the 1840s, they were the most active collectors in southern Africa.

They worked closely with the two Cape patrons of the period—German apothecary turned tobacco merchant Carl Ferdinand Heinrich von Ludwig and Irish botanist William Henry Harvey—who acted as important conduits through which Cape material and knowledge passed onward to Europe.^42^ Presumably after news had spread that *Hydnora* specimens had entered Europe in the early 1830s (as we will see later), Ludwig received requests from Berlin, Glasgow, and Dublin, imploring colonial physician Ludwig Pappe and employing Zeyher to fulfill European *desiderata* lists.^43^ However, it took a little longer than anticipated to collect another scientifically viable *Hydnora* specimen. Eventually, Pappe extracted some from Worcester in the vicinity of the Hex River, promptly dried them, and placed one in a preserving agent.^44^ Intended for William Jackson Hooker, renowned Professor of Botany at the University of Glasgow and later Director of Kew Gardens, and in consolation for his patience, Ludwig transmitted to him another rare parasitic plant in spirits: the *Ichthyosma wehdemanii*.^45^ On a visit to London, Harvey was able to view this exact specimen of *Ichthyosma*, exclaiming, “what a wonderful plant! I long to cut him up. These are very rare at the Cape, but the Aphyteja [*Hydnora*] are not quite so rare, and may be obtained from three or four places every season. I hope to get a good series.”^46^ In 1837, Harvey wrote to Hooker that he had “messengers flying in […] for Aphyteja and mean to have others soon so that I hope to own plenty of specimens—Ichthyosma I shall try for, but it comes from a very distant part of the colony.”^47^ These requests not only forced collectors to push further outside of the Cape's borders, but they began to create a value system ranking parasitic plants against one another based on rarity and geographical difficulty. As a “boundary object,” *Hydnora*'s meaning changed as it traversed between professional and geographical contexts.

At precisely the point in which there was an influx of desirable Cape plants circulating in Europe, access to certain parts of southern Africa became restricted. In any attempt to gather *Hydnora* specimens, former Kew Gardens collector James Bowie suggested that another potential *Hydnora* species could be “found at the frontiers” near the Fish River and Grahamstown, which he claimed to have seen on previous travels.^48^ As an employee of Ludwig and Harvey, Zeyher was asked to seek out this new varietal as well as more examples of *Ichthyosma*.^49^ The outbreak of the Sixth Xhosa War (1834–1836), however, disrupted Zeyher's attempts. Ludwig elaborated his displeasure about “the unexpected, sudden & formidable irruption of our barbarous neighbours, the Caffres” which jeopardized his ability to send any specimens “unless they are driven back and pursued by the Military and Burgher forces into the very heart of their savage country.”^50^ Yet again, Ludwig had confidence in his collectors to overcome these logistical difficulties. He ensured that Zeyher would do everything in his power to follow the burgher forces into the interior to “collect whatever he can find interesting” despite the danger presented by Xhosa raids.^51^ After the conclusion of the conflict, Zeyher attempted to drive north into “Massilicatres [*sic*] territory, a chieftan of part of the Zoola [*sic*] tribe.”^52^ After several Boer families were murdered “by that horrible despot,” he could scarcely persuade his African assistants to continue on, forcing him to retreat to the Orange River “where civilization commenced.”^53^ As a result, Ludwig and Harvey recognized that “any trip beyond the boundary is now out of the question.”^54^ Bowie offered another explanation as to why collectors faced difficulties during this period, claiming that “the wanton destruction of shrubs by the vagrant emancipated slaves & the consequent accumulation of sand on denuded grounds” better described the disappointment in unfulfilled requests.^55^ Here, Africans and their impenetrable environment are unjustly blamed for the pause in the physical and intellectual extension of European knowledge. This reveals a paradox: African assistants were both indispensable to finding plants like *Hydnora* and responsible for Europeans’ inability to source the plants they desired.

Though we know these collectors were dependent upon African knowledge and assistance, they also relied on the asymmetrical power structures and human exploitation that were built into the fabric of Cape society.^56^ This period was extremely turbulent, resulting in the fragmentation and displacement of numerous African communities and political units, forcing many Africans into the developing colonial labor market where they were easily exploited with low wages and manual labor by white farmers in the Western Cape and beyond. Thus, Africans and their labor should be understood more within the Cape's violent imposition of colonial systems of authority and the legacies of slavery during the Dutch period, rather than the dynamic and fluid hierarchies we see in work on intermediaries in South and Southeast Asia.^57^ The writings of Ecklon, Zeyher, and Drège reflect this. Their attitudes generally fall into place alongside white farmers’ prioritization of control, pejorative language, and sometimes physical violence, as they too often complained about the imbalance between rate of pay and perceived amount of work completed. Drège's brother Carl Friedrich often fixated on drunkenness.^58^ After hiring an African assistant named Wilm, Drège noted, “for a few blows I gave Wilm since he was drunk again, he ran to Vreede Richter to accuse me. Wilm was stripped of his contract to serve me longer.”^59^ Drège lamented a year later, “the people plague us a lot with drinking. Hans David chased out of service. Jacob Esau and Johan Cobus Bastert registered in court. Jacob Esau placed in prison because of drunkenness and brutality.”^60^ Eventually, African labor in these natural history outfits was gradually supplanted (although not wholly) by white farmer friends and their sons. In their view, this would be most cost‐effective for their enterprise: they would have “less annoyance” and more time for insect collecting, in Drège's words.^61^ Because their mindset was already economically frugal and commercially motivated, their thinking aligned neatly into the traditional exigencies of the colonial system, demonstrating the relationship between violence and natural history collecting.

## 3 Classification in the Metropole

The lack of *Hydnora* herbarium material across Europe sparked enormous debate, manifesting in questions of how botanists could classify plants without seeing them *in situ* and what characteristics should be considered in classifying flowers that “appeared decidedly afloral.”^62^ This process was initiated by Scottish botanist Robert Brown, keeper of the Banksian Botanical Collection at the British Museum in London, when he formally introduced the Sumatran parasite *Rafflesia arnoldii* to the scientific world in 1820.^63^ Appointed as naturalist to Matthew Flinders’ circumnavigation of Australia between 1801 and 1803, he subsequently became one of Britain's most important botanists and, notably, a fierce advocate of the natural system of classification. Although initially drawn to unassuming flora like cryptogams and mosses, he quickly turned his attention to the puzzling field of plant parasitism.^64^ In his 1821 inaugural report, he referred to the dearth of material evidence needed to better understand what he considered a necessary field of botanical research, claiming “sufficient materials, indeed, for such an investigation are hardly to be expected in collections, in which the parasite is most frequently separated from the root; and even when found in connection with it, is generally in a state too far advanced to afford the desired information.” Nonetheless, based on the fragments at his disposal, he posited that *Rafflesia* appeared most nearly allied to the European *Asarina* and the Mediterranean *Cytinus* on the one hand, and to *Hydnora* of southern Africa on the other, proposing that these groups potentially formed a so‐called natural family.^65^ In attempting to understand how the flower reproduced and its connection to other root parasites, Brown seemed unsure of his conclusions.

As botany began to develop into a distinct discipline, it became clear that many of the plants arriving from around the globe folded uncomfortably into the Linnaean system. A continued reliance on classifying plants by the number of pistils and fertile stamens meant that unrelated plants were placed together in many cases, making it increasingly difficult to establish kinships between plant groups.^66^ In recognition of this, botanists became embroiled in a debate: should they keep the one‐character method of Linnaeus or was it possible to use more than one morphological characteristic to differentiate when naming a new genus and species?^67^ Determining how to categorize plants like *Rafflesia* and *Hydnora* became part of this timely discussion. As botanists began to think more about the complex process of plant parasitism, it became impossible to use the Linnaean system to link plants together into families. They needed the natural system, which focused on the morphology, cell structure, reproduction, and anatomy of plants, and established an order of importance among plant features into a hierarchy.^68^ This helped to bring out the similarities between plants that would have otherwise been classified separately in the Linnaean system. The question for botanists then became: how important is parasitism as a plant feature? As botanists began to unravel this question, it was essential to make comparisons to other plants within and outside of southern Africa. One botanist described the identification of natural families as a “discovery that transcended geographical borders”; using the natural system opened a pathway for botanists to begin making global comparisons of plants.^69^ Plant classification therefore became a question about complex traits rather than simple features, and about global connections rather than local considerations. The question of parasitism forced botanists to reevaluate established concepts and work across disparate spaces.

Questions around materiality and knowledge transfer regarding *Hydnora* were not just present in the *in situ* attempts to collect botanical specimens from parts of southern Africa. Rather, these complications became even more pronounced the further *Hydnora* specimens traveled away from their natural environment. When Ecklon and Drège arrived to sell their specimens in Europe in 1833/1834, they attempted to capitalize on their unique finds in German scientific journals. In the Berlin‐based journal *Linnaea*, Ecklon was quick to announce the sale of his enormous collection, citing *Hydnora* specifically as one of the more “remarkable plants” collected on his travels.^70^ At the same time, Drège's patron (botanist Ernst Meyer) published a treatise describing *Hydnora* in relation to the initial eighteenth‐century assessments of Thunberg and Acharius in the journal of the German National Academy of Sciences Leopoldina.^71^ He also used it as an opportunity to discuss the discovery of a new species: *Hydnora triceps*.^72^ In a summary of the treatise in the same year's issue of *Linnaea*, an important point was made which was meant to bring some clarification to lingering questions about *Hydnora*. Citing it as “a member of the family of the Asarines, thus being neither a fungus nor a monocotyledonous plant,” the anonymous author sought to discredit the earlier hypotheses made by the Swedish botanists of the eighteenth century while also incorporating some of the recent work of Robert Brown.^73^ While demonstrating that *Hydnora* was not a fungus, the assumption that it belonged to “the Asarines” now became lodged into the classificatory imagining of the plant. If we think back to the meeting of German naturalists at the beginning of the paper, it is clear that Kunze was using what was the most up to date literature on *Hydnora* at that point. Still, understanding where such a plant fit into existing classificatory frameworks was difficult. Because most botanists had not traveled to Uppsala or London to view wet or dried *Hydnora* specimens in person, it would have been impossible to say otherwise, even with illustrations. Without strong material evidence to examine the differences between “the Asarines” and *Hydnora*, metropolitan botanists were forced to speculate.

In a paper read to the Linnean Society in 1834, Brown revised his classification of *Rafflesia* after the arrival of new specimens and *in situ* observations.^74^ It had been observed that “the discovery of the wonderful giant flower of India […] has brought to light several works […] [on these] miraculous formations without having given any detailed explanations about them or their relationship with similar root parasites.”^75^ Brown included new findings not just on *Rafflesia*, but on *Hydnora* and *Cytinus* too, discussing their affinities and whether they should be classed together. Because of the new specimens that had entered the market from Ecklon, Zeyher, and Drège, as well as Meyer's treatise, Brown could now claim that there were points in *Hydnora*'s structure “which seem to throw some light on one of the most difficult questions respecting *Rafflesia*,” bringing him to more fully realized conclusions about the nature of parasitism.^76^ At the meeting in Jena in 1836, German botanists also debated the issue of parasitism using the physical *Hydnora* example at their disposal.^77^ One believed that parasitism arose from the structures of the plants themselves. Another suggested parasitism occurred when a part of the plant was scratched, causing a particular kind of reaction to take place. A third argued that there was a parasite seed that germinated outside of the body of the plant. A final commentator seemed to think that some parasitic plants like *Lathraea* had a level of attraction—“it sucks, so to speak.”^78^ These continental botanists were working hard to try to get to the root of root parasitism. Ultimately, the scientific dialogue about parasitism was a transnational one: how should Europeans make sense of this physiological process and place it into an ordering system—one that was not equipped for this difference—and how should they order affinities of different root parasites from around the world?

However, once Brown finally published his 1834 paper eleven years later, he faced a challenge from British botanist William Griffith, his former student who had been working in scientific positions across the British Empire, primarily in India. Griffith published a multi‐part response questioning Brown's methodology and central premise, likewise invoking *Hydnora* and *Cytinus* to explain his findings.^79^ Drawing on his own *in situ* experience, his contentions were both taxonomic and material, and the latter wholly informed the former. As Jim Endersby maintains, drying plant material was “a uniform process of transforming plants into specimens in which, at each stage of the process, aspects of the original plant were destroyed.”^80^ Removing idiosyncrasies also eliminated the individuality of the plant, making it more difficult for metropolitan botanists to identify the singular characteristics that would have made it easier for them to understand how these plants parasitized other plants. It is also possible that Griffith was trying to lend credibility to the work of practitioners in the field like himself, who were often viewed as inferior from the metropole. Nonetheless, he believed that parasitism alone could hardly be used as the singular point of comparison between what he perceived as widely divergent plants.^81^

Griffith's key point was that determining characteristics in such a complicated process as parasitism could only be studied using live plants, or at least freshly cut ones. This had been forwarded by botanist Franz Unger in 1840, who suggested that the work of collectors in the field was absolutely essential for studying parasitism: “it will also make known in detail about their way of life, which must be studied on the spot.”^82^ Many botanists agreed; this method was seen as “more natural than experimental studies in a garden, and more accurate than the herbarium observation of dried specimens.”^83^ Thus, the herbarium specimens of *Rafflesia* available to Brown presented, in Griffith's opinion, incomplete evidence.^84^ Based on his own fieldwork rather than herbarium samples, he concluded that *Rafflesia* and *Hydnora* were not similar in their parasitism.^85^ Yet Griffith could not claim virtue, as he had never seen *Hydnora* in its natural habitat to make his own comparisons. Acting somewhat hypocritically in his opposition to Brown, the specimens of *Hydnora* he examined were “both in the dry state and in […] pyroligneous acid,” very likely the examples that Pappe had collected years earlier.^86^ The questions that arose from attempts to create order out of seeming chaos led Griffith into a lengthy reflection critiquing the system of botanical taxonomy, a process mainly conducted in the elite confines of the herbarium and museum. Where to study traits like parasitism—in the field or in the herbarium—seemed irreconcilable to practitioners in both spaces, and *Hydnora* was a central part of that conversation. This debate reflects something deeper about the insecurity of who was intervening in scientific debates on complex problems, and how practices of doing, question‐posing, explanation‐seeking, perceiving, and larger‐scale structure‐finding ideals were shifting in response to new material data.

As *Hydnora* moved through different hands, spaces, institutions, and ontologies, questions about agency were posed by those who worked with it. The defiance of plants like *Hydnora* and *Rafflesia* to submit to human interference seemingly contributed to the difficulty in studying their parasitism and internal structures. Zeyher certainly understood *Hydnora* in those terms, calling it one among “many a Cape plant” which was “obstinate in the first instance, to submit to the treatment and wishes of man.”^87^ For Brown and Griffith, the impenetrability of *Rafflesia* was tied to their understanding of its tropical home in the Sumatran rainforest. Like many unusual and exotic plants, it became deeply entwined with configurations of the environment. Yet, botanists dwelled very little on the landscapes which gave life to *Hydnora*, omitting it from grandiose portrayals and popular imaginings of the idiosyncrasy of nature. The scientific descriptions of *Hydnora*, and the illustrations which followed, omitted the plant's very specific ecological context so necessary in understanding how its parasitism functioned. This was part of the standardization process: uniform dried specimens and illustrations created a widely understandable system that could cross national and institutional contexts yet separated plants from their ecologies and cultural contexts. The enigmatic character of *Hydnora*, though, was not lost on those who studied it. Reactions to the flower's strangeness is best embodied by botanist C.G. Nees von Esenbeck 1825 in a hanging quote attached to Unger's *Beiträge zur Kenntniss der Parasitischen Pflanzen*: “Aphyteja Hydnora thus stands as a hieroglyphic key between two worlds, which like dream and waking, are laid out in an endless interrelationship and flee before us.”^88^

## 4 Conclusion

Plants have become an important tool for answering historical questions about the role (and agency) of non‐human actors in human‐centered stories. In this case, using *Hydnora* as a “boundary object” has offered a way of flattening the field, allowing us to investigate different nation‐states and imperial units, marginalized actors and spaces, and shifting epistemologies. Most importantly, it has helped to address some of the major themes debated by historians of science about the nature of scientific practice. In this case, the most obvious are around “circulation,” how natural objects and the knowledge produced about them might travel (or fail to travel) across land and sea, and the significant role of global communication in the process of making parasitism knowable.^89^ As a complex plant characteristic, attempts to understand parasitism reflected the deep concern about knowledge production felt by those in the higher echelons of European science. Identities were formed through collecting, classification, and colonialism in the rapidly changing world of nineteenth‐century science, but with this came increasing professionalization which increasingly separated the social worlds of colony and metropole. Only a select few would remain in control of how knowledge was produced, exerting power across the world from their metropolitan institutions.

The process of understanding plant features like parasitism was only possible with a more widespread acceptance of the natural system, the emergence of biogeography, and work on plant morphology, all of which were central to the practice of natural history in the German states but were only partially acknowledged or applied elsewhere in the early nineteenth century. This perhaps helps to explain why the crux of the parasitism debate happened between Robert Brown and German botanists: Brown had access to a vast supply of global plant specimens and was, for a time, one of the only British botanists utilizing all three simultaneously in his research. However, collectors and botanists working in colonial contexts made valuable contributions with their *in situ* experience, long overlooked because of the hierarchies of professionalization. They noticed patterns in the attributes and distribution of species which sometimes more closely resembled what would become plant ecology in the twentieth century, focusing on vegetation types, environmental factors, and ecological interactions. Though they had the same aims, they approached them with different views. Working “in the field” would become increasingly more significant for understanding plants and their relationships to the environment, inverting the distinctions between metropolitan and colonial knowledge that had so dominated how botany was practiced in the nineteenth century.^90^ Therefore, following *Hydnora* in this particular moment captures the tensions of shifting scientific ideas and scientific roles in a time of rapid imperial expansion.

While these are often human stories about allegedly heroic scientists, big scientific debates, and colonial violence, what they were fundamentally concerned with was plants, the stuff with which they made their reputation. The moment these collectors detached *Hydnora* from its roots, an indigenous African plant was both materially and intellectually abstracted to fit it into European notions of scientific knowledge. In attempts to understand the essence of parasitism, *Hydnora* both resisted and facilitated scientific understanding.

## Footnotes

1 Translation: The Society of German Naturalists and Physicians. N.A. 1836–1837, on 132. Worcester is located approximately 120 km from Cape Town.

2
Outram 1999.

3
Star and Griesemer 1989, on 393. See also: Star 2010.

4
Star and Griesemer 1989, on 413. See also: Bowker and Star 1999.

5
Trompette and Vinck 2009.

6
Star and Griesemer 1989, on 389.

7 Ibid.; Latour 1987.

8
Alberti 2005, on 567. See also: Bennett 2019 and Bowker 2025.

9
Cooley et al. 2023, on 1.

10
Appadurai 1986; Pearson 2015.

11
Bennett 2010, on 31–32.

12
Dürbeck et al. 2015, on 122.

13
Pearson 2016; Rees 2017; Saha 2021; Roy 2017; Skotnes‐Brown 2023.

14
Kimmerer 2013; Endersby 2016; Ayers 2019; Schiebinger 2004. Also, see the new journal *Plant Perspectives* from White Horse Press.

15
Lawrence 2022, on 631; Bishop 2018, on 8; Marder 2013.

16
Ayers 2019, on 338.

17 Among the abundant literature, see: Subramaniam 2024; Schiebinger and Swan 2005; Miller and Reill 1996; Camerini 1996; Secord 1994a; Secord 1994b; Massey 2003; McGregor 2018; Camerini 1997; Driver 2005.

18
Ayers 2019, on 416; Shinozuka 2022.

19
Nemser 2017.

20
Kohler 2007, on 441.

21
Endersby 2016; Ayers, 2019; Whittingham 2009.

22
Browne 1996; Dettelbach 1996.

23
van Wilgen 2016.

24 The infamous Table Mountain alone has more species of plants than the whole of the British Isles.

25
Visser 1981.

26 See: Thorogood 2018.

27
Westwood 2010, on 227.

28
Musselman and Visser 1989.

29
Bolin et al. 2009; Marloth 1905–1907, on 467.

30 Carl Ferdinand Heinrich von Ludwig to William Jackson Hooker, 27 August 1836, Royal Botanic Gardens, Kew (henceforth RBGK), DC 59/194. After this point, I will also shorten names that have been fully written once.

31 William Guybon Atherstone to Hooker, 4 March 1856, RBGK, DC 59/10. Here we wrestle with racist terminology. Terms such as “Bushman” or “Kaffir” are reproduced only in direct quotations, reflecting the actors’ categories.

32 However, Mkala et al. 2021 argues that traditional uses of *Hydnora* are poorly documented and need further research. See also: Williams et al. 2011, on 474; Hatt 2021.

33 This remains the type species of the *Hydnora africana* today. Bolin et al. 2011, on 255.

34
Svedelius 1944, on 130–131.

35
Mottram 2021, on 27; Thunberg 1777, on 144–148. Apparently, he later corrected this in a letter to Linnaeus: N.A. 1781, on 352.

36
von Linné and Acharius 1776. *Aphyteja* was one of the original names given to *Hydnora* by Linnaeus.

37 Famous visitors include the naturalists Hinrich Lichtenstein, Francis Masson, and William Burchell.

38 See: Mackinney 2024; Bregman 2006; Easterby‐Smith 2018.

39 Carl Friedrich Drège collected zoological and entomological specimens in partnership with his brother. See also: Delfs 2024.

40
Mackinney 2022; Coote et al. 2017.

41
Kinukawa 2011, on 166.

42 A former apothecary, Ludwig married the widow of a wealthy tobacco merchant. He pursued natural history in his leisure time, donated money to Cape scientific institutions, and ran a privately‐owned botanic garden (1829–1848). William Henry Harvey was the Treasurer General of the Cape (1836–1842) until he took up the post of curator of the Trinity College Herbarium (1844) and Professor of Botany of the Royal Dublin Society (1848).

43 William Henry Harvey to Hooker, 16 December 1835, RGBK, DC 58/46; Harvey to Hooker, 4 February 1836, RGBK, DC 58/47; Harvey to Hooker, 5 June 1836, RGBK, DC 58/49; Ludwig to Hooker, 3 April 1836, RGBK, DC 58/191.

44 Ludwig to Hooker, 27 January 1835, RGBK, DC 58/188.

45 This is now known as *Sarcophyte sanguinea*. Von Schlechtendal 1828, on 194–198.

46 Harvey to Hooker, 16 June 1836, RBGK, DC 58/50.

47 Harvey to Hooker, 6 October 1837, RBGK, DC 58/59.

48 Ludwig to Hooker, 27 August 1836, RGBK, DC 58/194. Grahamstown is the former colonial name of now Makhanda. Bowie is perhaps referring to another parasitic plant—the *Cytinus sanguineus—*which was, at one point, thought to be related to (though different from) *Hydnora*, named the *Aphyteïa multiceps*. Burchell 1822, on 213.

49 Harvey to Hooker, 6 October 1837, RGBK, DC 58/59; Ludwig to Hooker, 28 February 1835, RGBK, DC 58/190.

50 Ludwig to Stewart Murray, 28 February 1835, RBGK, DC 58/189.

51 Ibid.

52 Karl Zeyher to Hooker, 25 October 1840, RBGK, DC 58/238. Zeyher has attempted to pronounce Mzilikazi, ruler of the rising Ndebele polity, phonetically. If he was attempting to travel north of the Vaal River, that placed Zeyher in what is now the Transvaal region near the end of the *mfecane* and as the Voortrekkers began to arrive in the region. The clashes between Mzilikazi and the Voortrekkers ultimately pushed Mzilikazi's amaNdebele into Matebeleland (southwestern Zimbabwe). See: Eldredge 2014; Wright 2009. For revisionist analyses of Zulu‐centrism in this period, including the possibility of abandoning the term *mfecane* as part of the outdated Zulu‐centric line of argument: Hamilton 1995.

53 Zeyher to Hooker, 25 October 1840, RBGK, DC 58/238.

54 Harvey to Hooker, 29 June 1838, RBGK, DC 58/66.

55 James Bowie to Hooker, 12 March 1842, RBGK, DC 58/12.

56 For example: Keegan 1996.

57
Schaffer et al. 2009; Fan 2004.

58
Crush and Ambler 1992; Lawrance et al. 2006.

59 National Library of South Africa (henceforth NLSA), Drège Family Collection, MSC 61.8.526, 5 May 1830.

60 NLSA, Drège Family Collection, MSC 61.8.526, 2 July 1831.

61 NLSA, Drège Family Collection, MSC 61.1.103, 7 March 1831.

62
Ayers 2019, on 374.

63 See: Ayers 2024.

64 Ibid., on 375. See also: Barnard 2015.

65
Brown 1821, on 225; Ayers 2019, on 373–374.

66
Kury and Albuquerque 2021, on 41.

67
Stevens 1994. See also: Miracle 2021; Müller‐Wille 2013.

68
Kury and Albuquerque 2021, on 41.

69 Ibid., on 55–56.

70
N.A. 1833, on 183–184; Ecklon 1833, on 391.

71
Meyer 1833.

72 The *Hydnora triceps* is found exclusively in Namaqualand and southern Namibia (Drège's specimen was collected from the Okiep area of the northwestern Cape) and was thought extinct until the 1980s. Maass and Musselman 2004, on 1; Tennakoon et al. 2007.

73
N.A. 1833, on 183.

74 This paper was also influenced by an intervention from botanist Karl Ludwig Blume on the *Rafflesia patma*. Blume introduced a new species of *Rafflesia* (*Rafflesia patma*) in his *Flora Javae*, classing them with other large flowering parasites (*Rhizantheæ*) and renouncing the idea that *Rafflesia* might be a fungus or other cryptogam. Blume 1828–1851; Nees von Esenbeck and Daniel 1825.

75
von Schlechtendal 1827, on 671–673.

76
Brown 1845, on 221.

77 While it is difficult to know to what extent they had engaged with Brown's work, Brown had attended the *Versammlung deutscher Naturforscher und Ärzte* in Bonn (1835). His name is listed under the attendees in: N.A. 1836, on 654. In a French report on the Bonn meeting, we can see that Brown discussed his new findings on *Rafflesia* and *Hydnora*. N.A. September 1836, on 191.

78
N.A. 1836–1837, on 160.

79
Griffith 1845. Some biographical information on Griffith in: Axelby 2008, on 155–157.

80
Endersby 2008, on 82.

81
Griffith 1845, on 304–305.

82
Unger 1840, on 15.

83
Bonneuil 2002, on 194.

84
Ayers 2019, on 390.

85
Griffith 1845, on 313.

86
N.A. 1845, on 192.

87 Zeyher to Hooker, 5 March 1851, RBGK, DC 59/351.

88 Translation: Contribution to the Knowledge of Parasitic Plants. 96, on 14. Robert Mitchell argues that the Romantics underwent “a vertiginous falling for the strange and dark life of vegetation,” which seems characteristic of the sentiment Nees von Esenbeck is attempting to convey. Mitchell 2010.

89
Gänger 2017; Schiebinger 1998.

90
Tilley 2011.
